# Risk factors associated with onset of medication-related osteonecrosis of the jaw in patients treated with denosumab

**DOI:** 10.1007/s00784-021-04261-4

**Published:** 2021-11-23

**Authors:** Alexander Wick, Philipp Bankosegger, Sven Otto, Bettina Hohlweg-Majert, Timm Steiner, Florian Probst, Oliver Ristow, Christoph Pautke

**Affiliations:** 1 Department of Oral and Maxillofacial Surgery and Facial Plastic Surgery, University Hospital, LMU Munich, Lindwurmstr. 2a, 80336 Munich, Germany; 2 Department of Oral and Maxillofacial Surgery, University of Halle, Ernst-Grube-Straße 40, 06120 Halle, Germany; 3 Medizin & Aesthetik, Clinic for Oral and Maxillofacial and Plastic Surgery, Lenbachplatz 2a, 80333 Munich, Germany; 4 Department of Oral and Maxillofacial Surgery, University of Aachen, Pauwelsstraße 30, 52074 Aachen, Germany; 5 Department of Oral and Maxillofacial Surgery, University of Heidelberg, Im Neuenheimer Feld 400, 69120 Heidelberg, Germany

**Keywords:** MRONJ, Osteonecrosis of the jaw, Risk factors, Bisphosphonates, Denosumab

## Abstract

**Objectives:**

While risk factors of bisphosphonate (BP) associated osteonecrosis of the jaw have been properly analyzed, studies focusing on risk factors associated with denosumab (DNO) are sparse. The purpose of this study was to identify risk factors influencing the onset of medication-related osteonecrosis of the jaw (MRONJ) in patients receiving antiresorptive treatment (ART) with DNO by comparing patients suffering from MRONJ and patients without MRONJ. Multiple variables were evaluated including the impact of a previous BP intake.

**Materials and methods:**

A retrospective single-center cohort study with patients receiving DNO was conducted. One-hundred twenty-eight patients were included and divided into three groups: I (control, *n* = 40) receiving DNO with absence of MRONJ; group II (Test 1, *n* = 46), receiving DNO with presence of MRONJ; and group III (Test 2, *n* = 42) sequentially receiving BP and DNO with presence of MRONJ. Patients’ medical history, focusing on the identification of MRONJ risk factors, was collected and evaluated. Parameters were sex, age, smoking habit, alcohol consumption, underlying disease (cancer type, osteoporosis), internal diseases, additional chemo/hormonal therapy, oral inflammation, and trauma.

**Results:**

The following risk factors were identified to increase MRONJ onset significantly in patients treated with DNO: chemo/hormonal therapy (*p* = 0.02), DNO dosage (*p* < 0.01), breast cancer (*p* = 0.03), intake of corticosteroids (*p* = 0.04), hypertension (*p* = 0.02), diabetes mellitus (*p* = 0.04), periodontal disease (*p* = 0.03), apical ostitis (*p* = 0.02), and denture use (*p* = 0.02). A medication switch did not affect MRONJ development (*p* = 0.86).

**Conclusions:**

Malignant diseases, additional chemotherapy, DNO dosage, and oral inflammations as well as diabetes mellitus and hypertension influence MRONJ onset in patients treated with DNO significantly.

**Clinical relevance:**

Patients receiving ART with DNO featuring aforementioned risk factors have a higher risk of MRONJ onset. These patients need a sound and regular prophylaxis in order to prevent the onset of MRONJ under DNO treatment.

## Introduction

Medication-associated osteonecrosis of the jaw (MRONJ) is a rare but severe adverse side effect of an antiresorptive therapy (ART). Initially described as being associated with the intake of bisphosphonates (BP) [1, 2], it became obvious that other antiresorptive drugs such as denosumab (DNO) have also the risk of inducing MRONJ [3–5]. Increasing tumor incidences, longer patient survival, adjuvant antiresorptive therapy strategies, and rising numbers of first-line regimes for osteoporosis are turning MRONJ into a disease of increasing importance [6–8].

Interestingly, both the pharmacological mechanisms and pharmacokinetics of BP and DNO are markedly different: BP are admitted orally or intravenously and accumulate in bone by selectively binding to hydroxyapatite. In the soluble phase when released from bone in an acidic milieu, BP molecules interfere intracellularly in the mevalonate pathway and inactivate various cell types but in particular osteoclasts [9]. DNO, in contrast, is a human monoclonal antibody that selectively binds to the RANK ligand, a key cytokine for the differentiation, maturation, and activation of osteoclasts. Subcutaneously admitted DNO is inactivated by the immune system within weeks as other allogenic antibodies and does not accumulate in the body.

Both drugs have an osteoclast suppressing effect in common. Thus, conditions in which osteoclast activity is essentially needed (such as local bone inflammation processes of the jaws or bone remodeling after tooth extraction) may not be overcome and can lead to an osteonecrosis. While this pathogenesis theory is widely accepted [10–12] there are additional risk factors associated with MRONJ onset. Risk factors for BP therapy have previously been described in several studies (see Table 1). To date, however, only limited data with small sample sizes are available focusing on the impact of certain risk factors associated with DNO intake [13].

**Table 1:** Availability of data concerning risk factors associated with MRONJ in patients treated with DNO compared to BP and sequentially with both drugs

	**Variable**	**Denosumab references**	**Denosumab/bisphosphonate reference**	**Bisphosphonate references**
**Demographic factors**	Sex	[13]	[14]	[15, 16]
	Age	[13]	*	[15, 16]
	Smoking habit	[13]	*	[15, 17–19]
	Alcohol consumption	[13]	*	[15, 17–19]
**Medical comorbidities**	Cancer type	[13, 14]	[14]	[15, 16, 18–23]
	Osteoporosis	[13, 14]	[14]	[15, 16, 18, 20, 24]
	Diabetes mellitus	[13]	[14]	[15, 16, 18, 20, 25]
	Diseases of cardio-vascular system	*	*	[16, 18]
	Hypertension	*	[14]	[16, 18, 21]
	Renal disease	*	*	[16, 18]
	Gastrointestinal disease	*	*	[16, 18]
	Thyroid malfunctions	*	[14]	[16, 18]
	Rheumatic diseases	*	[14]	[16, 18, 22]
	Infectious diseases	*	*	[16, 18, 22]
**Co-medication associated with underlying disease**	Chemotherapy	[13]	[14]	[1, 15, 22, 24, 26]
	Molecular targeted therapy	[13]	[14]	[1, 15, 18–20, 22, 24, 26]
	Corticosteroid therapy	[13]	[14]	[1, 18–20, 22, 24, 26]
	Hormonal therapy	[13]	*	[1, 15, 22, 24, 26]
**Dental comorbidities**	Extraction	[14]	[14]	[1, 15–25, 27–29]
	Periodontal disease	[13]	[14]	[15–18, 21, 27]
	Apical ostitis	[13]	[14]	[15, 17, 20, 23, 27, 30]
	Retained root	*	*	[15, 17]
	Dental cysts	*	*	[15, 17, 30]
	Dental implants	[31]	[14]	[15–17, 23, 32–34]
	Endodontic treatment	*	*	[35, 36]
	Use of dentures	[13]	[14]	[15–18, 21, 27]
	Trauma	*	*	[37, 38]
	Poor oral hygiene	*	[14]	[15–18, 20, 21, 23, 27]

^*^To our knowledge, few data (mostly case reports or studies with small cohorts) exist concerning these risk factors. Of note, the sample size of [13] was *n* = 14

Identification of risk factors could improve prophylaxis and prevention of MRONJ. Therefore, the purpose of this study was to find out other risk factors inducing the onset of MRONJ in patients receiving antiresorptive treatment with DNO.

The authors hypothesize that (I) certain demographic, co-medications, and oral-health factors influence the onset of MRONJ; (II) the switch from a previous BP intake to DNO intake furthermore influences the onset of MRONJ; and (III) that the presence of dental implants at the start of ART elevates the risk of MRONJ onset.

The specific aims of this study were to estimate (I) which factors, derived from patients medical history, influence MRONJ onset; (II) whether a combination of DNO/BP treatment elevates the risk of MRONJ onset; and (III) whether the presence of dental implants would impact MRONJ incidence.

## Patients and methods

This study was approved by the local medical association authority (Bayerische Landesärztekammer, ethic committee number: 2020–1228) and was carried out according to the Declaration of Helsinki 7th revision of 2013.

### Study design

We designed and implemented a retrospective single-center cohort study and consecutively enrolled a sample derived from the source population of subjects who were treated at the Department “Medicine and Aesthetics,” Clinic for Oral and Maxillofacial Surgery, Munich, between 2011 and 2020, and fulfilled a predefined selection protocol with the following inclusion criteria: (1) antiresorptive therapy (ART) with at least one or more doses of DNO in patients’ medical history, (2) presence of MRONJ (for groups II and III) according to criteria for MRONJ according to the American Association of Oral and Maxillofacial Surgery (AAOMS) [39], (3) availability of panoramic dental X-ray, (4) follow-up examinations of at least 12 months, and (5) complete medical history including co-medication.

The exclusion criteria were as follows: (1) history of irradiation in the head and neck region, (2) metastatic bone disease of the maxillofacial region, (3) missing follow-up examinations, (4) missing panoramic dental X-ray, and (5) missing information in the medical history.

**Fig. 1:**
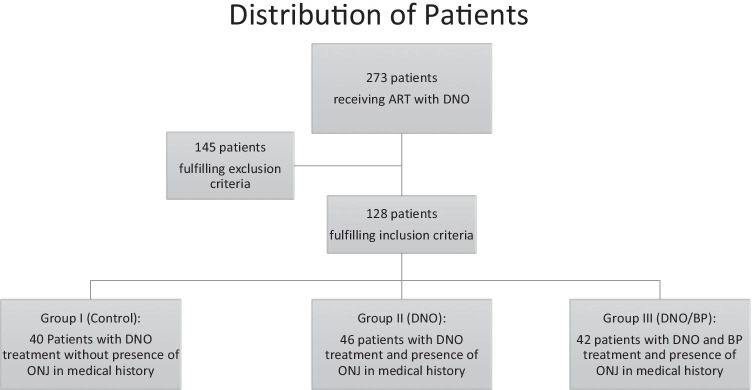
Flowchart of patient distribution into three groups

All patients fulfilling inclusion criteria were consecutively divided into three groups, according to patient’s medical history: group I (control), DNO treatment and no presence of MRONJ; group II (Test 1, DNO), DNO treatment with presence of MRONJ; and group III (Test 2, DNO/BP), switch of ART from BP to DNO with presence of MRONJ (see Fig. 1).

### Study variables

The study variables were as follows: presence of MRONJ in patient’s medical history (yes/no), demographic factors (age at last clinical examination, sex, duration of follow-up, smoking habits, and alcohol consumption), underlying disease requiring ART (cancer type and/or osteoporosis, bone metastasis, and time since initial diagnosis of underlying disease), ART type of medication (duration of medication and medication switch), co-medication associated with underlying disease (chemotherapy or molecular targeted therapy, corticosteroid therapy, hormonal therapy, and antiangiogenic therapy), MRONJ (time since initial diagnosis of MRONJ, location, and surgical therapy), additional medical history (hypertension, diabetes mellitus, diseases of cardio-pulmonary system, thyroid malfunctions, rheumatic diseases, and infectious diseases), and intraoral findings (number of teeth, retained root, presence of dental implants prior to ART, periodontal disease, apical periodontitis, dental cysts, denture use, and presence of current dental procedure in area of MRONJ) (see Table 1).

### Primary and follow-up examinations

Patients not yet treated with DNO were referred to Medicine and Aesthetics by different regional oncologists and osteologists, prior to ART, for primary examination to assess oral health status. Patients’ medical records on underlying disease, ART, medical comorbidities and co-medications, and oral findings were evaluated. Additionally, a panoramic X-ray was performed, and mucosal integrity and periodontal diseases were evaluated according to the European Federation of Periodontology guidelines [40].

With absence of MRONJ these patients underwent yearly (12 months) follow-up examinations undergoing the same examination. If required prophylactic treatment was performed according to the 2018 German MRONJ guidelines [41] and consisted of extraction of non-preservable and periodontally compromised teeth and dental implants, the excision of dental cysts and foreign bodies, and the reduction of denture-associated sore spots.

Patients with onset of MRONJ were referred to Medicine and Aesthetics either by the same oncologists and osteologists or by their dentists initially discovering exposed bone or MRONJ lesions. Most patients presenting MRONJ received surgical treatment following the same therapeutic standardized protocol as recently described [42]. Follow-up examinations in cases of MRONJ were conducted quarterly (3 months). All examinations were conducted by the same investigators (CP, BHM, AW, PB). Surgery was performed by one and the same oral and maxillofacial surgery specialist (CP). When extraction of non-preservable or periodontally compromised teeth and dental implants was necessary under ongoing DNO therapy, procedures were again performed accordingly to the 2018 German MRONJ guidelines [41]. Treatment consisted essentially of minimally invasive techniques with consecutive smoothing of sharp bone edges, complete mucoperiostal closure, and perioperative antibiotic therapy (amoxicillin/clavulanic acid 875/125 mg 1–0-1, in case of allergy: clindamycin 600 mg 1–1-1, starting 1 week preoperatively and continuing for up to 3 weeks postoperatively) as well as a weekly follow-up in the first 4 weeks.

There was no recommendation for discontinuation of ART (drug holiday) by the investigators of this study, but in few cases (see Tables 1 and 2) ART was paused by the prescribing osteologists and/or oncologists at the time of first consultation in our clinic.

**Table 2:** Patient characteristics and demographics

	**Variables**	***n* (%) or median [range]**
**Demographic factors**	Male	49 (38.3%)
	Female	79 (61.7%)
	Age (years)	72.2 [61.9–80.1]
	Duration of follow-up (months)	14.0 [12.7–20.2]
	Smoking habit	24 (18.8%)
	Alcohol consumption	21 (16.4%)
**Underlying disease**	Osteoporosis	57 (44.5%)
	Cancer	86 (67.2%)
	Breast	40 (31.3%)
	Prostate	31 (24.2%)
	Other	15 (11.7%)
	Bone metastasis	86 (67.2%)
	Time since initial diagnosis of underlying disease (years)	9.0 [6.0–14.3]
**Antiresorptive therapy**	Denosumab	128 (100%)
	120 mg	78 (60.9%)
	60 mg	50 (39.1%)
	Bisphosphonates	42 (32.8%)
	Zoledronate	21 (16.4%)
	Ibandronate	14 (10.9%)
	Other	7 (5.5%)
	Duration of medication (months)	38.7 [22.9–59.2]
**Co-medication associated with underlying disease**	Chemotherapy or molecular targeted therapy	37 (28.9%)
	Corticosteroid therapy	7 (5.5%)
	Hormonal therapy	24 (18.8%)
	Antiangiogenic therapy	5 (3.9%)
**Osteonecrosis of the jaw**	Presence of MRONJ	88 (68.8%)
	Time since initial diagnosis of MRONJ (years)	3.0 [1.7–5.1]
	Location of MRONJ	Upper jaw: 31 (32.0%)
		Lower jaw: 66 (68.0%)
	Drug holiday	13 (10.2%)
	Surgical therapy	81 (92.0%)
**Medical history**	Hypertension	43 (33.6%)
	Diabetes mellitus	8 (6.3%)
	Diseases of cardio-pulmonary system	49 (38.3%)
	Thyroid malfunctions	29 (22.7%)
	Rheumatic diseases	10 (7.8%)
	Infectious diseases	1 (0.8%)
**Intraoral findings**	Number of teeth	2211 (100%)
		20.0 [11.3–25.0]
	Upper jaw	10.0 [4.0–12.0]
	Lower jaw	11.0 [6.0–13.0]
	Retained root	5 (3.9%)
	Patients with dental implants prior to ART	34 (26.6%)
	Number of dental implants	139 (6.3%)*
	Periodontal disease	88 (68.8%)
	Apical periodontitis	48 (37.5%)
	Dental cysts	5 (3.9%)
	Denture use	52 (40.6%)
	Dental procedures	60 (46.8%)

^*^Percentage measured according to absolute number of teeth

### Data acquisition and analysis

Data acquisition was performed retrospectively and derived from patients’ medical records in a yes/no manner according to the previously performed primary and follow-up examinations (see Table 2).

Statistical analysis was performed with SPSS Version 21 (SPSS Inc., Chicago, IL, USA) as well as R Version 4.0.3. Initially all non-nominal data were analyzed for normal distribution applying a Kolmogorov–Smirnov test.

The statistical differences between the three groups were evaluated using *T*-test, Chi-square test, and ANOVA or Kruskal–Wallis test with post hoc analysis applying a Bonferroni test. Analysis of certain risk factors for the development of MRONJ was furthermore conducted by applying logistic regression.

### Patient cohort and characteristics

In total, 273 individuals were identified as having received ART with DNO, 145 patients met one or more exclusion criteria and were excluded from this study (see Fig. 1), and 128 patients met the inclusion criteria. The median follow-up on 31 December 2020 (end of data acquisition) was 14.0 [12.7–20.2] months with a maximum follow-up time of 80 and a minimum of 12 months (see Table 1).

Most of the patients in our cohort suffered from stage IV cancer (of which breast and prostate cancer were the prominent malignant diseases) followed by osteoporosis. All cancer patients had at least one or multiple skeletal metastases. The median time since initial diagnosis of underlying disease was 9 years.

Patients received most frequently DNO in the dosage 120 mg in 60.9%, 37 patients (28.9%) patients received chemotherapy or molecular targeted therapy, 24 patients (18.8%) were treated with a hormonal therapy. Seven patients (5.5%) received an additional corticosteroid therapy, 5 patients (3.9%) received antiangiogenic therapy, and 42 patients (32.8%) had received ART with BP before DNO treatment (see Fig. 1).

The main variable MRONJ was present in 88 patients (68.8%) with a median time since initial diagnosis of MRONJ of 3 years. MRONJ was found in the upper jaw in 31 cases (32.0%), in 66 cases (68.0%) in the lower jaw, and in 9 patients (7.0%) in both jaws. Surgical therapy of MRONJ lesions was performed in 81 cases (92.0%). A discontinuation of ART at the time of the dental procedure was performed in 13 cases (10.2%). Of note, the investigators of the study gave no recommendation on drug holiday. The ART prescribing physician initiated the drug holiday.

The most frequent co-morbidities were diseases of cardio-pulmonary system hypertension in 49 patients (38.3%), followed by arterial hypertension in 43 patients (33.6%), thyroid malfunctions in 29 patients (22.7%), rheumatic diseases in 10 patients (7.8%), and diabetes mellitus in 8 patients (6.3%).

Intraoral findings presented a total number of 2211 teeth with a median of 10 in the upper and 11 in the lower jaw. Five patients (3.9%) had a retained root, 88 patients (68.8%) revealed moderate to severe periodontal disease, 48 patients (37.5%) had apical periodontitis, 5 patients (3.9%) had dental cysts, and 52 patients (40.6%) were using removable dentures. In 60 patients (46.8%) dental surgical procedures were performed. A total of 139 dental implants prior to ART were found in 34 patients (26.6%) with a median of 3 implants per patient (see Table 2).

The cohort was divided into three groups dependent on the presence of MRONJ and the antiresorptive medication:

- **Group I (control): *n* = 40** 15 males (37.5%) and 25 females (62.5%) with a median age of 69.8 years. Three patients (6.6%) had died until the end of data acquisition.
- **Group II (Test 1, DNO): *n* = 46** patients with 49 MRONJ lesions, 20 males (43.5%) and 26 females (56.5%) with a median age of 75.1 years. Four patients (9.5%) had died until the end of data acquisition.
- **Group III (Test 2, DNO/BP): *n* = 42** patients with 48 MRONJ lesions, 14 males (33.3%) and 28 females (66.7%) with a median age of 70.8 years. Two patients (4.4%) had died until the end of data acquisition.

To further display patient characteristics and demographics of patients with MRONJ onset and those with MRONJ absence, Table 3 demonstrates parameter distribution in groups I, II, and III.

**Table 3:** Comparison of patient characteristics and demographics within groups

*n (%) or median [range]*				
	**Variables**	**Group I (control) (*n* = 40)**	**Group II (Test 1, DNO) (*n* = 46)**	**Group III (Test 2, DNO/BP) (*n* = 42)**
**Demographic factors**	Male	15 (37.5%)	20 (43.5%)	14 (33.3%)
	Female	25 (62.5%)	26 (56.5%)	28 (66.7%)
	Age (years)	69.8 [61.3–80.4]	75.1 [63.5–83.8]	70.8 [61.8–76.3]
	Duration of follow-up (months)	13.6 [12.5–20.9]	13.3 [12.8–18.2]	16.2 [13.4–24.1]
	Smoking habit	4 (10.0%)	9 (19.6%)	11 (26.2%)
	Alcohol consumption	4 (10.0%)	8 (17.4%)	9 (21.4%)
**Underlying disease**	Osteoporosis	27 (58.7%)	14 (27.5%)	16 (34.8%)
	Cancer	19 (41.3%)	37 (72.5%)	30 (65.2%)
	Breast	7 (36.8%)	15 (40.5%)	18 (60.0%)
	Prostate	9 (47.4%)	13 (35.1%)	9 (30.0%)
	Lung	0	2 (5.4%)	0
	Kidney	0	3 (8.1%)	0
	Other	3 (15.8%)	4 (10.8%)	3 (10.0%)
	Bone metastasis	19 (41.3%)	37 (72.5%)	30 (65.2%)
	Time since initial diagnose of underlying disease (years)	6.5 [3.5–10.5]	8.2 [5.1–12.0]	10.5 [8.6–15.1]
**Antiresorptive therapy**	Denosumab	40 (100%)	46 (100%)	42 (100%)
	120 mg	13 (32.5%)	35 (76.1%)	30 (71.4%)
	60 mg	27 (67.5%)	11 (23.9%)	12 (28.6%)
	Bisphosphonates	*No BP*	*No BP*	42 (100%)
	Zoledronate			21 (50.0%)
	Alendronate			5 (11.9%)
	Ibandronate			14 (33.3%)
	Other			2 (4.8%)
	Duration of medication (months)	35.8 [18.5–47.6]	35.3 [23.4–58.1]	47 [24–66.9]
**Co-medication associated with underlying disease**	Chemotherapy or molecular targeted therapy	7 (17.5%)	18 (39.1%)	16 (38.1%)
	Corticosteroid therapy	0	6 (13.0%)	2 (4.8%)
	Hormonal therapy	2 (5.0%)	13 (28.3%)	9 (21.4%)
	Antiangiogenic therapy	0	1 (2.2%)	4 (9.5%)
**Osteonecrosis of the jaw**	Presence of MRONJ	*No MRONJ*	46 (100%)	42 (100%)
	Time since initial diagnosis of MRONJ		2.6 [1.8–4.3]	3.7 [1.7–5.6]
	Location of MRONJ		Upper jaw: 15 (30.6%)	Upper jaw: 16 (33.3%)
			Lower jaw: 34 (69.4%)	Lower jaw: 32 (66.7%)
	Surgical therapy		42 (91.3%)	39 (92.9%)
	Drug holiday	8 (20.0%)	3 (6.5%)	2 (4.8%)
**Medical history**	Hypertension	8 (20.0%)	21 (45.7%)	16 (38.1%)
	Diabetes mellitus	0	6 (13.0%)	2 (4.8%)
	Diseases of cardio-pulmonary system	12 (30.0%)	20 (43.5%)	17 (40.5%)
	Thyroid malfunctions	9 (22.5%)	15 (32.6%)	5 (11.9%)
	Rheumatic diseases	4 (10.0%)	3 (6.5%)	3 (7.1%)
	Infectious diseases	1 (2.5%)	0	0
**Intraoral findings**	Number of teeth	24 [13.3–26.8]	19 [7.3–24]	20 [12–23]
	Upper Jaw	11 [5–14]	8 [4–11]	9.5 [5.3–12]
	Lower Jaw	12 [8.5–14]	10 [4.3–12]	10.5 [6–12]
	Retained root	0	1 (2.2%)	4 (9.5%)
	Dental implants prior to ART	3 [2.0–6.3]	2.5 [2–3.8]	4 [3–5]
	Periodontal disease	22 (55.0%)	35 (76.1%)	31 (73.8%)
	Apical periodontitis	12 (30.0%)	18 (39.1%)	23 (54.8%)
	Dental cysts	2 (5.0%)	1 (2.2%)	2 (4.8%)
	Denture use	10 (25.0%)	27 (58.7%)	15 (35.7%)
	Dental procedures	18 (45.0%)	22 (47.8%)	20 (47.6%)

## Results

To assess which parameters influence the onset of MRONJ in patients treated with DNO, each of the above mentioned parameters was initially compared with the parameter “presence of ONJ” (Yes/No) applying chi-square and *T*-tests.

Statistically significant differences in patients with MRONJ absence (control) and patients with MRONJ presence (groups II and III) were found in the parameters underlying disease (*p* = 0.01), DNO dosage (*p* < 0.01), co-medications (chemotherapy, hormonal therapy, etc.) (*p* = 0.05), vascular diseases (*p* = 0.04), and dental inflammations.

**Fig. 2:**
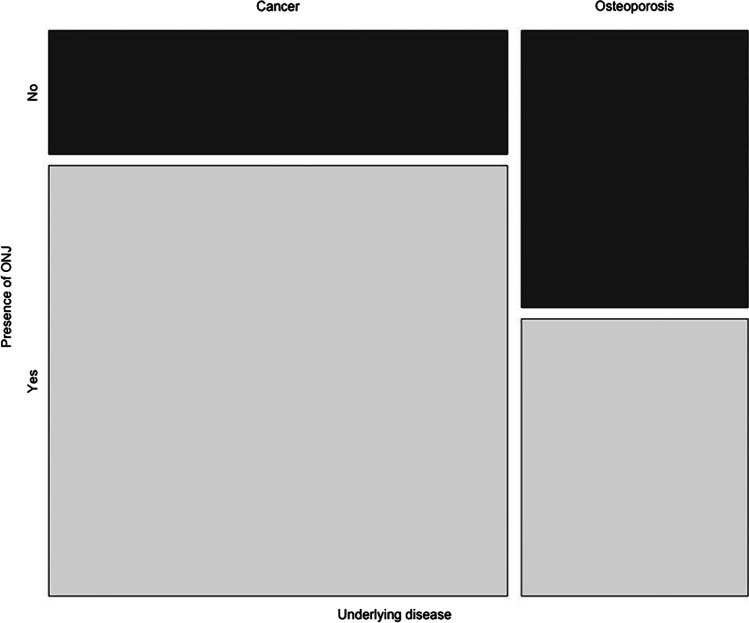
Mosaic plot: underlying disease vs. presence of MRONJ

**Fig. 3:**
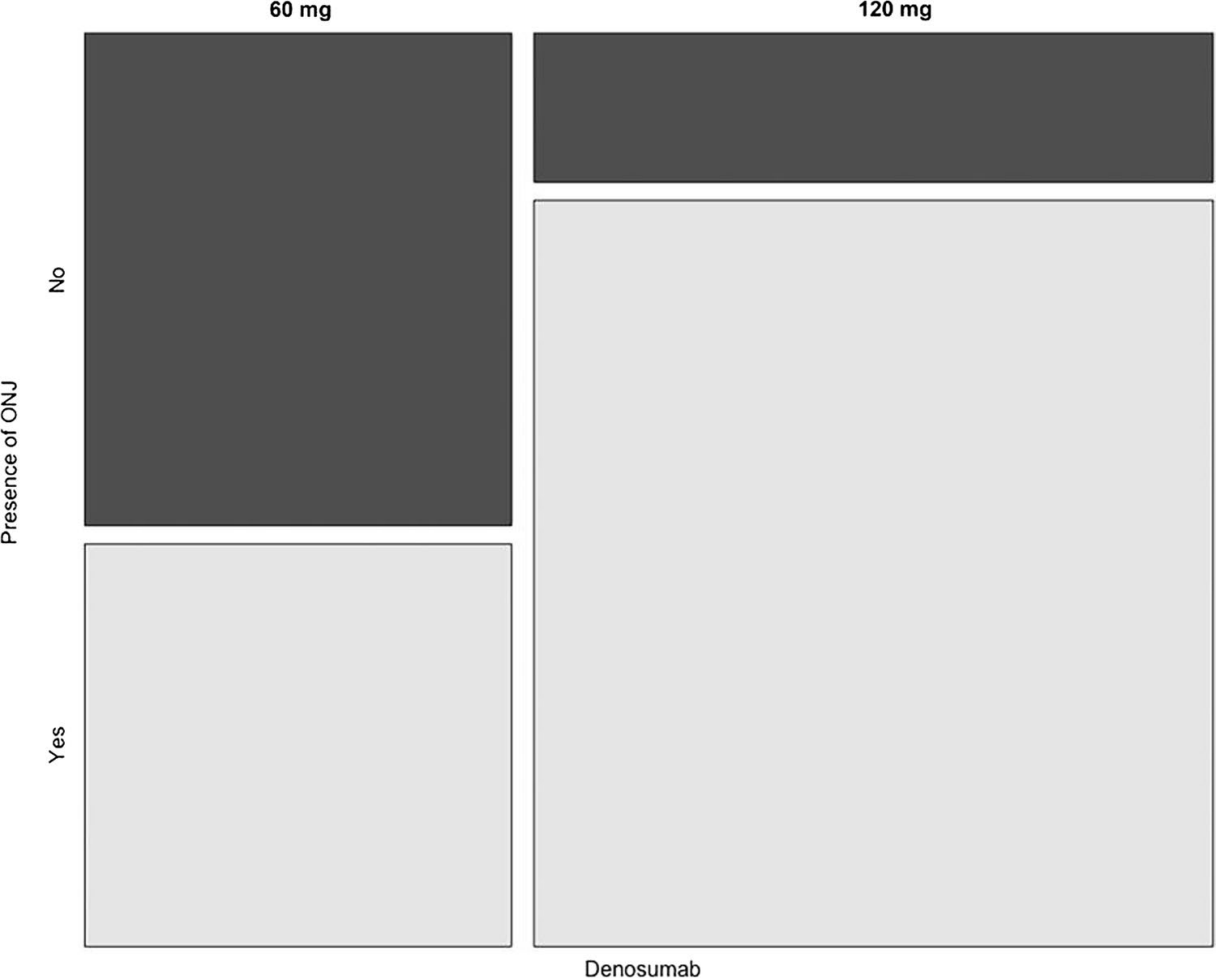
Mosaic plot: DNO dosage vs. presence of MRONJ

Exemplary the parameters underlying disease and DNO dosage are displayed as mosaic plots. Patients suffering from cancer (*n* = 86) as underlying disease showed a significantly higher presence of MRONJ (*p* < 0.01) compared to patients suffering from osteoporosis (*n* = 56) (see Fig. 2). Patients receiving high-dose (120 mg) DNO (*n* = 78) presented more onset of MRONJ compared to patients receiving low-dose (60 mg) DNO (*n* = 50) (see Fig. 3).

**Fig. 4:**
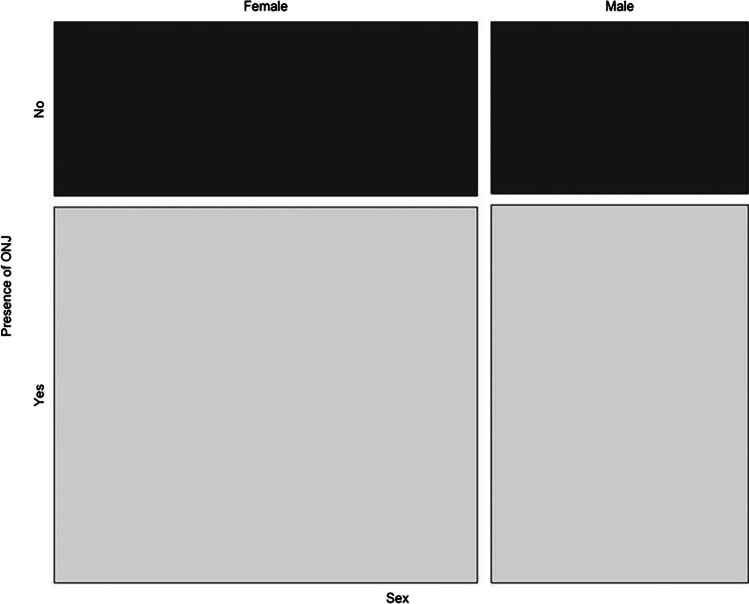
Mosaic plot: sex vs. presence of MRONJ

Even though there were significantly more women (*n* = 79) than men (*n* = 49) included in this study, the variable “sex” was not identified as a risk factor and MRONJ onset was distributed equally for both genders (see Fig. 4).

To further define risk factors, these previously identified parameters were analyzed performing logistic regression. The variable duration of ART (OR 1.07 [1.00–1.13], *p* = 0.04), breast cancer (OR 2.83 [1.12–7.12], *p* = 0.03), chemotherapy or molecular targeted therapy (2.97 [1.18–7.46], *p* = 0.02), hormonal therapy (6.33 [1.41–28.43], *p* = 0.02), hypertension (2.96 [1.22–7.16], *p* = 0.02), periodontal disease (2.46 [1.12–5.40], *p* = 0.03), apical ostitis (2.04 [0.92–4.51], *p* = 0.02), and use of dentures (2.74 [1.20–6.28], *p* = 0.02) presented significant predictors of MRONJ onset with an odds ratio larger than 1 (see Table 4).

**Table 4:** Risk factors for MRONJ onset in logistic regression analyses

Variables	Odds ratio [CI]	*p-Value*
Duration of ART	1.07 [1.00–1.13]	0.037
Breast cancer	2.83 [1.12–7.12]	0.027
Chemotherapy	2.97 [1.18–7.46]	0.021
Hormonal therapy	6.33 [1.41–28.43]	0.016
Hypertension	2.96 [1.22–7.16]	0.016
Periodontal disease	2.46 [1.12–5.40]	0.026
Apical ostitis	2.04 [0.92–4.51]	0.018
Denture use	2.74 [1.20–6.28]	0.017

*CI* confidence interval, *p* significance

The variables drug holiday (OR 0.24 [0.07–0.79], *p* = 0.02) and presence of dental implants prior to ART (0.85 [0.189–1.018], *p* = 0.04) however were identified to decrease the risk of MRONJ onset in this study (see Table 5).

**Table 5:** Factors decreasing the risk of MRONJ in logistic regression analyses

Variables	Odds ratio [CI]	*p-Value*
Drug holiday	0.241 [0.073–0.792]	0.019
Dental implants prior to ART	0.846 [0.189–1.018]	0.043

*CI* confidence interval, *p* significance

## Discussion

The results of this study revealed that higher DNO dosage, additional chemotherapy, hormonal therapy and corticosteroid therapy, breast cancer as underlying disease, co-morbidities like hypertension and diabetes mellitus, periodontal disease and apical periodontitis, and the use of dentures elevate the risk of MRONJ onset in patients treated with DNO. A minor effect was also associated with prolonged intake of DNO.

When comparing the afore mentioned risk factors for onset of MRONJ in patients treated with DNO and those previously described for onset of MRONJ in patients treated with BP (see Table 1) it becomes clear that these findings correlate well. Considering the different pharmacokinetics and pharmacodynamics of BP and DNO, this underlines the importance of inflammatory processes as the main triggering events [11, 15] in the development of MRONJ.

### Risk factors

For BP it was shown that co-medications such as chemotherapy or corticosteroid therapy alongside local risk factors, e.g., dental procedures, apical ostitis, and periodontal disease, presented a significant impact on the onset of MRONJ [15, 23, 39, 43]. The female gender as well as breast cancer as an underlying disease were also identified as having a predilection for MRONJ [44–48].

In a recent study patients under DNO therapy with absence and presence of MRONJ were compared [13]; however, the sample size with *n* = 14 was limited. The number of patients in the present study was considerably higher and an additional investigation group was added: patients under antiresorptive therapy with a medication switch from BP to DNO and the presence of MRONJ. The three investigated groups presented statistically significant differences in regard to the underlying disease, the dosage of DNO, co-medications, additional diseases, and intraoral findings.

The number of patients suffering from stage IV cancer was significantly higher in both MRONJ groups (DNO and DNO/BP) compared to the control group whose population predominantly suffered from osteoporosis. This result is concordant to the dosage of DNO distributed among the groups: high-dose DNO (120 mg) was mainly found in the MRONJ groups, whereas low-dose DNO (60 mg) was the main DNO derivate in the control group with no onset of MRONJ. The results of this study suggest that higher doses of DNO elevate the risk of MRONJ.

Co-medications such as chemotherapy, hormonal therapy, and corticosteroid therapy pose a significant risk to the development of DNO associated MRONJ. This can be explained by the fact that these medications might lead to immunosuppression and thus, indirectly, alter the risk of local inflammation and consecutively the onset of MRONJ. Breast cancer as an underlying disease was also associated with an elevated MRONJ risk [48–50].

Hypertension and with less impact also diabetes mellitus as vascular diseases were also identified as risk factors. An impaired vascular system decreases the blood circulation of bone structures particularly of bones with exceptional blood supply as well as a high proportion of cortical structures such as the lower jaw [15] and thus possibly abating the onset of MRONJ.

Periodontal disease, apical periodontitis, and the use of dentures were shown to elevate the risk of MRONJ. This is concordant with the theory of inflammation as a key factor in the pathogenesis of MRONJ. On the one hand mucosal inflammation due to periodontal diseases and denture-associated sore spots pose a continuous stimulus on mucosal integrity. Micro-lesions in mucosa and periodontal apparatus thus enable oral bacteria to penetrate into the bone and cause local inflammation [51]. Due to the reduced osteoclast activity under ART the defense ability towards bone infections is markedly reduced. These observations support the hypothesis that local inflammations are of paramount importance in the pathogenesis of MRONJ [18, 25, 52].

Even though regular consumption of alcohol and smoking did not pose significant risk factors for MRONJ in this study, they are clear risk factors for oral diseases, e.g., periodontal disease and impaired wound healing, thus indirectly favoring the development of MRONJ [53].

These findings correlate well with those of the few other studies we found. Okuma et al. [13] furthermore showed that prolonged DNO intake and sex influence onset of MRONJ. In this study prolonged intake of DNO did only present a mild risk of MRONJ. It is however a logical consequence that longer DNO application adds to the likelihood of triggering events taking place. This is in line with the findings of other studies demonstrating that prolonged intake of DNO significantly increased the development of MRONJ [38, 54]. As for sex, it is readily assumed that gender may present a risk factor; however, it needs to be considered that mainly female patients are affected by breast cancer and osteoporosis [22] and thus shift the balance of patients receiving ART to the female gender.

### Dental implants

Taking a closer look at dental implants as a risk factor for increased MRONJ onset in patients treated with BP results are ambiguously, if not contrary. In a recent overview evaluating several studies on the impact of dental implants on the onset of MRONJ, dental implants were identified as a risk factor [55]. The same conclusion was drawn from another working group by Jacobsen et al. [56] showing that dental implants were risk factors for MRONJ onset in patients treated with BP, whereas in a Korean cohort, these results could not be confirmed by Ryu, Kim, and Kwon [31].

To our knowledge, the impact of dental implants in patients treated with DNO has been scarcely investigated [14] and was therefore a key finding of this study. When applying logistic regression (see Table 5) it was revealed that the presence of dental implants prior to ART did not increase but instead decrease the risk of MRONJ onset in patients treated with DNO. This finding could be related to a potentially better oral hygiene in patients caring for the dental implants compared to those without dental implants, as those patients with dental implants prior to ART presented fewer cases of periodontitis in this study. Whatever the reason may be, we can assert from this study that the presence of dental implants prior to ART does not impact onset of MRONJ negatively. Periimplantitis, as a risk factor for MRONJ, seems to be as significant as periodontitis and should be avoided just as much in patients at risk. A poor oral hygiene appears to have a significant impact on the development of MRONJ in patients treated with DNO just as it has been reported in those patients treated with BP [7, 15].

### Medication switch

The assumed impact of a medication switch from BP to DNO did not however influence MRONJ onset. When compared to one another, groups II (DNO) and III (DNO/BP) did not show an increased MRONJ risk or a significant difference in risk factors. A similar result has been reported in a previous study by the same investigators as of this study [42]. In afore mentioned study it was also shown that a medication switch did neither influence the time to onset of MRONJ nor the outcome after surgical MRONJ therapy negatively.

### Drug holiday

The number of patients receiving a drug holiday in this study was relatively low, and these findings should be evaluated with care. A finding of this study was that the risk of MRONJ could be reduced by more than 75% in patients undergoing a drug holiday around dental procedures. A large prospective study would be advisable for future studies addressing this aspect.

The appliance of a drug holiday is widely discussed. Osteologists and oncologists [24, 57, 58] often advise not to pause ART even for a short term as the onset of skeletal related events (SRE) may yet occur. A meta-analysis of multiple studies concerning drug holidays has shown that a drug holiday does not reduce risk of MRONJ [55]. However, most studies in this meta-analysis focused on BP not on DNO. Two studies addressed the influence of a DNO drug holiday on the wound healing after the onset of MRONJ [14, 59]. Regarding the pharmacological differences between BP and DNO a short-termed DNO drug holiday surrounding a possible triggering event, like tooth extraction, could very well decrease the risk of MRONJ.

### Discontinuation of DNO

There was no recommendation from the authors to discontinue the antiresorptive therapy neither for BP nor for DNO. However, in several cases the ART was stopped by the prescribing oncologist/practitioner. Due to the fact that the pharmacological effect of denosumab attenuates within weeks there is a substantiation for a drug holiday. Nevertheless, the decision for a discontinuation should be an interdisciplinary one. There are no recommendations in the German guidelines for prostate carcinoma, breast cancer, or multiple myeloma for the discontinuation of ART with DNO [60–62].

### Limitations

The limitation of the present study is the small sample size evaluated. Due to the retrospective character of this study, inclusion in the study was defined after a predefined period of time. However, for further evidence the present data can be used for power estimations of a prospective trial in the future. Another limitation is the heterogeneous intake of antiresorptive medication, underlying disease, and immunomodulatory co-medications. Nevertheless, the application of multifactorial regression models (considering covariance as medication types and/or underlying disease) should be the aim in subsequent confirmatory studies. Of note, it will be increasingly difficultly to find pure intervention groups in the future. This might be progressively part of the discussion when performing MRONJ studies. However, we tried our best to create large and comparable study groups, all of whom were operated on by the same surgeon and followed regular check-ups.

## Conclusion

In conclusion, the results of this study revealed statistically significant correlations of the onset of MRONJ in patients treated with DNO in patients receiving higher DNO dosage, additional chemotherapy, hormonal therapy and corticosteroid therapy, those suffering from breast cancer, hypertension, diabetes mellitus, periodontal disease and apical periodontitis, and those using dentures. A minor effect was associated with prolonged intake of DNO.

These findings correlate well with risk factors associated with MRONJ onset in patients treated with BP. MRONJ onset is a multi-factorial event that occurs in patients receiving BP as well as those treated with DNO despite the medication’s different pharmacokinetic and pharmacodynamic mechanisms. This underlines the importance of inflammatory processes as the main triggering event in the development of MRONJ. Concluding that prophylactic treatment prior to DNO application as well as regular oral examinations and carefully performed dental procedures after DNO application are fundamental elements in MRONJ prevention and patients at risk need to be educated that good dental hygiene is paramount to reduce MRONJ development.
